# Taurine and inflammatory diseases

**DOI:** 10.1007/s00726-012-1361-4

**Published:** 2012-07-19

**Authors:** Janusz Marcinkiewicz, Ewa Kontny

**Affiliations:** 1 Department of Immunology, Jagiellonian University Medical College, 18 Czysta St., 31-121 Kraków, Poland; 2 Department of Pathophysiology and Immunology, Institute of Rheumatology, Warsaw, Poland

**Keywords:** Taurine, Taurine chloramine, Taurine bromamine, Inflammatory diseases, Rheumatoid arthritis, Neutrophils, Myeloperoxidase, Antioxidants

## Abstract

Taurine (2-aminoethanesulfonic acid) is the most abundant free amino acid in humans and plays an important role in several essential biological processes such as bile acid conjugation, maintenance of calcium homeostasis, osmoregulation and membrane stabilization. Moreover, attenuation of apoptosis and its antioxidant activity seem to be crucial for the cytoprotective effects of taurine. Although these properties are not tissue specific, taurine reaches particularly high concentrations in tissues exposed to elevated levels of oxidants (e.g., inflammatory cells). It suggests that taurine may play an important role in inflammation associated with oxidative stress. Indeed, at the site of inflammation, taurine is known to react with and detoxify hypochlorous acid generated by the neutrophil myeloperoxidase (MPO)–halide system. This reaction results in the formation of less toxic taurine chloramine (TauCl). Both haloamines, TauCl and taurine bromamine (TauBr), the product of taurine reaction with hypobromous acid (HOBr), exert antimicrobial and anti-inflammatory properties. In contrast to a well-documented regulatory role of taurine and taurine haloamines (TauCl, TauBr) in acute inflammation, their role in the pathogenesis of inflammatory diseases is not clear. This review summarizes our current knowledge concerning the role of taurine, TauCl and TauBr in the pathogenesis of inflammatory diseases initiated or propagated by MPO-derived oxidants. The aim of this paper is to show links between inflammation, neutrophils, MPO, oxidative stress and taurine. We will discuss the possible contribution of taurine and taurine haloamines to the pathogenesis of inflammatory diseases, especially in the best studied example of rheumatoid arthritis.

## Introduction

Acute inflammation is a physiological response of tissues to harmful stimuli such as pathogens, damaged cells or cancer cells and irritants. This response, mediated predominantly by innate immunity, is responsible for elimination of these injurious stimuli and for the subsequent healing process. The major cells involved in acute inflammation are neutrophils: phagocytes responsible for microbial killing and for generation of various proinflammatory mediators. The myeloperoxidase–halide system plays a unique role in killing pathogens phagocytosed by neutrophils (Klebanoff 1968, 2005) through generation of hypochlorous acid (HOCl), a potent microbicidal and cytotoxic oxidant (Thomas 1979). Remarkably, MPO is the only mammalian enzyme that oxidizes Cl^−^ into HOCl (Gaut et al. 2001). Moreover, MPO can also oxidize Br^−^ to produce hypobromous acid (HOBr) (Thomas et al. 1995). Upon contact with a pathogen, activated phagocytes (both neutrophils and macrophages) produce a respiratory burst characterized by intense uptake of oxygen. Oxidant production begins when a membrane-associated NADPH oxidase reduces molecular oxygen to superoxide, which then yields H_2_O_2_. In neutrophil phagolysosomes, myeloperoxidase (MPO) uses H_2_O_2_ to convert chloride ion to HOCl, or bromide ion to HOBr (Klebanoff 1968; Thomas 1979; Henderson et al. 2001)

$$ {\text{Cl}}^{ - } {\text{ + H}}_{ 2} {\text{O}}_{ 2} {\text{ + H}}^{ + } \to {\text{ HOCl + H}}_{ 2} {\text{O,}} $$

$$ {\text{Br}}^{ - } {\text{ + H}}_{ 2} {\text{O}}_{ 2} {\text{ + H}}^{ + } \to {\text{ HOBr + H}}_{ 2} {\text{O}} . $$

**Fig. 1:**
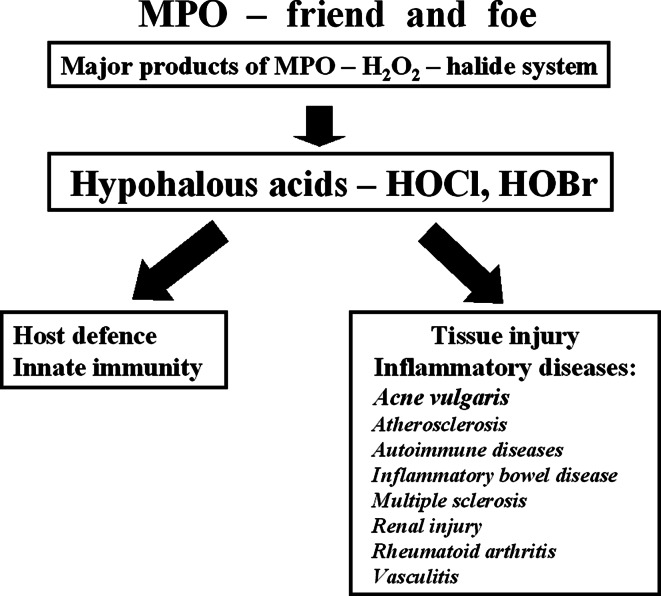
Hypohalous acids, the major products of PO–halide system exert both a beneficial (microbicidal) and detrimental (tissue injury) role in neutrophil-associated inflammation

Both hypohalous acids, HOCl and HOBr, are components of innate immunity and protect the host from infections by using their oxidizing potential to kill pathogens, but they may also damage host tissue. The microbicidal effects of HOCl have been linked to oxidation of methionine residues in bacterial cytosolic and inner membrane proteins (Rosen et al. 2009). On the other hand, overproduction of these oxidants and insufficient neutralization by antioxidants may lead to the development of oxidative stress and chronic inflammation (Smith 1994; Weiss 1988). Such a scenario may contribute to pathogenesis of inflammatory diseases, in which the neutrophil MPO–halide system is involved (Fig. 1). The above information clearly suggests that antioxidants play a crucial role in maintaining homeostasis and in amelioration of the harmful effect of oxidative stress. We asked the question whether taurine and/or taurine haloamines play a role in the pathogenesis of inflammatory diseases. We will focus on the role of TauCl in the regulation of inflammation in rheumatoid arthritis, the best studied diseases in our laboratories (Marcinkiewicz and Kontny 2012).

## Taurine links to inflammation and oxidative stress

**Fig. 2:**
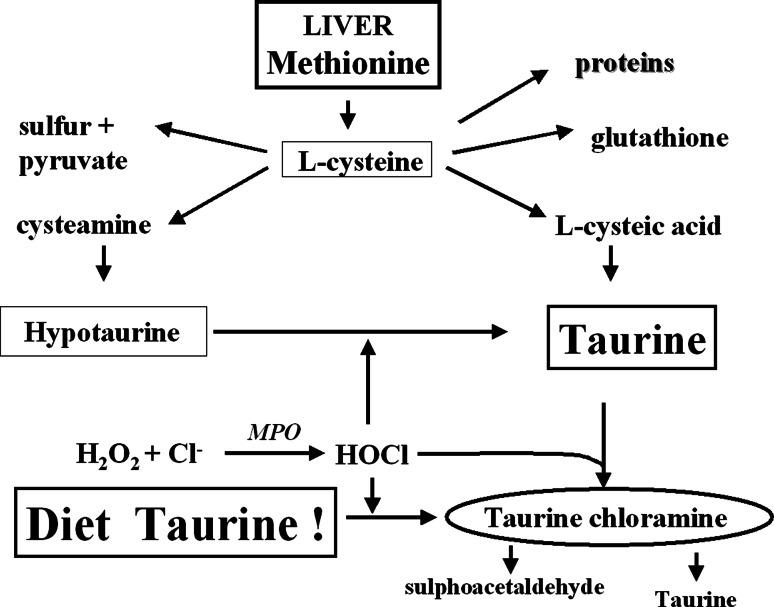
Fate of endogenous and diet taurine in activated neutrophils

Taurine, a semi-essential sulfur-containing β-amino acid, is present at high concentrations in most cells of all animal species (Sturmann 1993). In humans, taurine is formed from methionine and cysteine metabolism via hypotaurine in hepatocytes. Other cells (e.g., neutrophils) contain very high concentrations of taurine due to taurine uptake from the blood, a source of both endogenous and diet taurine (Fig. 2) (Bouckenooghe et al. 2006).

Taurine tissue distribution is characterized by low concentrations of taurine in the plasma and extracellular fluids (ranging from 10 to 100 *μ*M) and high intracellular concentrations of taurine reaching up to 50 mM, depending on the cell type (Huxtable 1992; Learn et al. 1990). The biosynthetic capacity of humans to produce taurine is limited in neonates (the effect amplified by prematurity) and also declines with aging and some pathological stages (trauma, sepsis). In these situations, the diet is likely to be an important taurine source (Redmond et al. 1998).

**Fig. 3:**
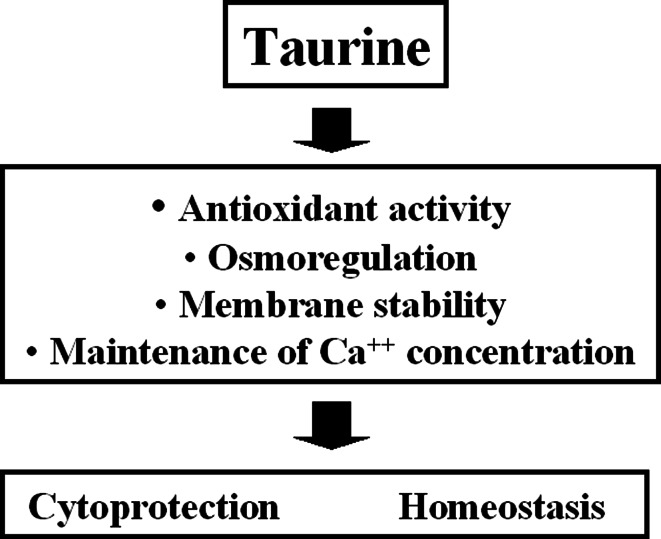
Biological functions of intracellular taurine and the immune cells

The high taurine levels in phagocytes and accumulation in taurine inflammatory lesions suggests its role in innate immunity (Schuller-Levis and Park 2004). Activated phagocytes generate a variety of microbicidal and toxic oxidants produced by the peroxidase system in these cells. As taurine is present at high concentrations in leukocytes, one may hypothesise that taurine deficiency will affect the immune cell functions. Indeed, prolonged taurine deficiency in cats leads to profound abnormalities in the immune system including significant leukopenia, a decreased respiratory burst in neutrophils and depletion of cells from B cell areas of lymph nodes and spleen (Schuller-Levis et al. 1990). However, there is no clear evidence concerning the association between taurine deficiency and a defect of the immune system in humans. On the other hand, it is commonly accepted that taurine plays an important role in the immune system as an antioxidant to protect cells, including leukocytes, from oxidative stress (Schaffer et al. 2009; Wang et al. 2009). Therefore, the primary role of taurine is cytoprotection and maintaining homeostasis of cells involved in acute and chronic inflammatory/oxidative stress (Fig. 3).

Oxidative stress is a major factor responsible for tissue damage in conditions such as infection, acute and chronic inflammation, cancer and aging. At a site of inflammation, oxidative stress is mediated by reactive oxygen species (ROS) generated primarily by activated leukocytes (neutrophils, macrophages, eosinophils). ROS play a beneficial role in host defense against pathogens, but they are also responsible for tissue injury (Weiss 1988; Smith 1994). A variety of antioxidants are involved in the prevention of oxidant-induced cell damage and reduction of oxidative modification of self-molecules, primarily high molecular compounds such as lipids, proteins and DNA. Antioxidants (the antioxidant network) act through one of three mechanisms: (1) reduction of ROS generation, (2) neutralization of ROS and (3) interference with the action of ROS (Schaffer et al. 2009).

**Fig. 4:**
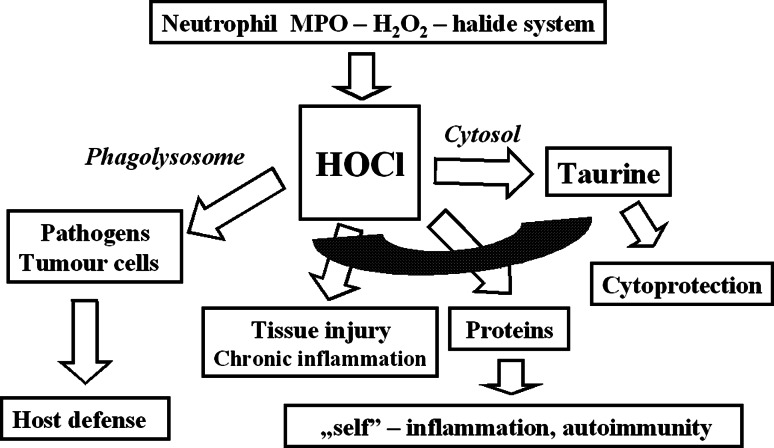
Targets of HOCl at a site of inflammation and its interaction with taurine. Inside phagolysosomes, HOCl kills ingested microbes. Outside phagolysosomes, taurine neutralizes detrimental effects of HOCl on neighboring cells and protects “self” molecules from oxidative modification. TauCl, the product of this reaction, is less toxic than HOCl. TauCl is not membrane permeable, oxidizes distinct targets and causes less damage to biologically active molecules (Marcinkiewicz and Kontny 2012)

Taurine is found at particularly high concentrations in tissues exposed to elevated levels of oxidants, suggesting its role in the attenuation of oxidative stress (Green et al. 1991; Jeon et al. 2009; Oliveira et al. 2010). Indeed, there have been numerous reports indicating taurine as an effective antioxidant, but the mechanism underlying its antioxidant activity remains unclear. The best established antioxidant action of taurine is neutralization of hypochlorous acid (HOCl), an extremely toxic oxidant generated by the MPO–halide system (Weiss et al. 1982). This activity explains the anti-inflammatory properties of taurine, as its reaction with HOCl results in generation of taurine chloramine (TauCl), a more stable and less toxic anti-inflammatory mediator (Weiss et al. 1982; Thomas 1979) (Fig. 4).

Therefore, taurine may be considered the component of innate immunity with a special impact on the development of acute inflammation. However, not all of the antioxidant actions of taurine are related to HOCl, because they can occur in systems lacking neutrophils. Although taurine is incapable of directly scavenging classical ROS, it has been suggested that it is an effective inhibitor of ROS generation. It has been shown that taurine enhances expression and activities of antioxidant enzymes, such as superoxide dismutase, catalase and glutathione peroxidase (Jang et al. 2009).

In conclusion, the data presented above suggest that the major role of taurine in the immune system is associated with taurine’s antioxidant properties, namely, with its ability to react with HOCl or HOBr to generate the biologically active but less toxic mild oxidants taurine chloramine (TauCl) and taurine bromamine (TauBr), respectively

$$ {\text{Taurine + HOCl}} \to {\text{taurine chloramine + H}}_{ 2} {\text{O,}} $$

$$ {\text{Taurine + HOBr }} \to {\text{taurine bromamine + H}}_{ 2} {\text{O}} . $$

## Taurine haloamines (TauCl, TauBr): a role in host defense

### TauCl and TauBr antimicrobial capacity

It is well known that oxidants generated by phagocytes at a site of inflammation are involved in host defense against microbes. Among them, hypohalous acids (HOCl, HOBr), extremely strong microbicidal agents, play a crucial role in killing of pathogens by neutrophils and eosinophils. They can kill a wide spectrum of Gram-positive and Gram-negative bacteria, fungi (yeast and molds), viruses, protozoa and worm larvae (Klebanoff 1968; Weiss 1988; Thomas et al. 1995). TauCl and TauBr, the physiological products of the MPO–halide system, show bactericidal, fungicidal, antiviral and antiparasitic properties, as demonstrated in vitro in a number of papers (Nagl et al. 2000b, 2001; Gottardi et al. 2005; Marcinkiewicz et al. 2000, 2006a; Yazdanbakhsh et al. 1987).

TauCl, the product of activated neutrophils, reaches micromolar concentrations (<100 μM) at the site of inflammation. At these physiological concentrations and at neutral pH, TauCl shows very weak antimicrobial activity. However, in acidic milieu which is typical for an inflammatory environment (pH 4–6), the ability of TauCl to kill pathogens increases significantly due to formation of the more potent TauCl_2_ (taurine dichloramine). TauCl_2_ is more bactericidal than TauCl, especially against Gram-negative bacteria, probably due to better penetration into bacteria. In addition, transfer of the active chlorine (transchlorination) from TauCl to amino groups of other molecules enhances its activity, mainly because of the formation of monochloramine (NH_2_Cl) (Gottardi et al. 2005; Gottardi and Nagl 2010; Nagl et al. 2003). However, it has not been proven whether endogenous TauCl also contributes to the killing of microbes in vivo. On the other hand, exogenous TauCl proved to be extremely well tolerated by human tissues and even 1 % aqueous solution of TauCl may be applied locally in the treatment of some skin and mucous infections (Gottardi and Nagl 2010).

TauBr, in contrast to TauCl, seems to be an effective microbicidal agent at very low physiologic concentrations <10 μM, even at neutral pH (Marcinkiewicz et al. 2006a, b). These results suggest that TauBr may contribute to host defense against microbes, although this still needs to be confirmed in vivo.

Importantly, the data investigating TauCl and TauBr antimicrobial activity in vitro have all been collected using planktonic form of bacteria, but not sessile bacteria hidden in a biofilm. Recently, there has been a tremendous interest in the role of biofilms in chronic infectious diseases and in the resistance of biofilms to antibiotics, disinfectants and phagocytosis (Costeron et al. 1991). As microbial biofilms are the most common mode of growth of bacteria and fungi in nature (O’Toole et al. 2000), it is reasonable to study whether TauCl and TauBr are able to kill bacteria hidden in a biofilm or destroy a protective exopolymeric matrix of growing biofilms. Our preliminary data suggest that taurine haloamines, especially TauBr, are promising candidates in the local therapy of biofilm-associated infections such as chronic sinusitis, otitis media, acne vulgaris and periodontal diseases.

### Anti-inflammatory properties of TauCl and TauBr

Acute inflammation is characterized by a massive neutrophil infiltration and generation of a variety of inflammatory mediators such as cytokines, eicosanoids and ROS (Weiss 1988; Thomas 1979). Studies from many laboratories have demonstrated that taurine haloamines (TauCl, TauBr) exert both bactericidal and anti-inflammatory properties (Park et al. 1993, 1995; Marcinkiewicz et al. 1995b, 1998, 2000, 2005; Quinn et al. 2003; Kim et al. 1996; Kim and Cha 2009). Taurine haloamines inhibit the production of proinflammatory cytokines (TNF-α, IL-1β and IL-6) (Marcinkiewicz et al. 1995a; Park et al. 1997; Barua et al. 2001). Moreover, it has been shown that TauCl reduces the production of nitric oxide (NO) and prostaglandin E_2_ (PGE_2_) and decreases the activity of matrix metalloproteinases (Chorazy-Massalska et al. 2004; Park et al. 2000; Kim et al. 2007). The above-mentioned anti-inflammatory properties together with the capacity of TauCl to induce leukocyte apoptosis suggest that TauCl may be involved in the resolution and termination of acute inflammation (Klamt and Shacter 2005). Interestingly, taurine, in contrast to TauCl, protects cells from apoptosis as shown in a number of in vitro studies (Jong et al. 2011; Maher et al. 2005).

### Impact of TauCl and TauBr on the induction of antioxidant network

**Fig. 5:**
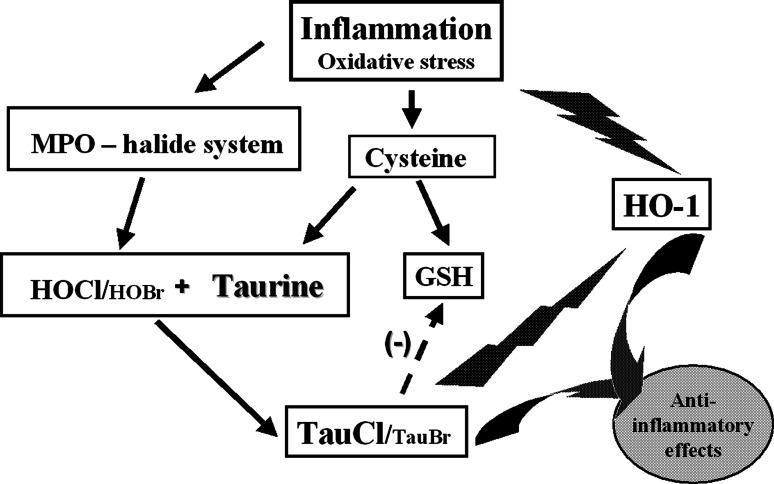
Association of taurine with the antioxidant network: a redundancy of the immune system. Taurine haloamines, the products of MPO–halide system, function as a physiological link between cysteine pathway and the heme-oxygenase-1 system (HO-1)

It is well documented that taurine protects cells against oxidative injury (Schaffer et al. 2009). In acute inflammation, which is characterized by neutrophil infiltration and generation of ROS by the MPO–halide system, taurine antioxidant activity is primarily related to the neutralization of HOCl and HOBr, as described above. It is also well known that TauCl and TauBr, apart from antimicrobial and anti-inflammatory properties, may also show antioxidant-like biological effects (Park et al. 1995; Marcinkiewicz et al. 1995b, 1998). TauCl and TauBr suppress the activity of phagocytic cells, thereby reducing their ability to consume oxygen and induce respiratory burst. In addition, TauCl reduces the production of ROS by increasing the expression of peroxyredoxin-1 and thioredoxin-1, the antioxidant enzymes normally induced by the activation of NF-E2-related factor-2 (Nrf2) (Kim et al. 2010a, b). Moreover, TauCl and TauBr, in a similar, dose-dependent manner, significantly enhanced in vitro the expression of heme-oxygenase-1 (HO-1) in various cells (Olszanecki and Marcinkiewicz 2004; Olszanecki et al. 2008; Kim et al. 2010a, b; Marcinkiewicz et al. 2009). The induction of HO-1 plays an especially important role in tissue homeostasis, as the products of HO-1-mediated heme degradation regulate important biological processes including oxidative stress and inflammation (Wagener et al. 2003). In addition, HO-1 reduces synthesis of proinflammatory heme proteins such as COX-2 and iNOS (Ryter et al. 2002). Therefore, one may speculate that at a site of inflammation, TauCl and/or TauBr will induce HO-1 in neighboring non-activated cells to protect them against oxidative stress (Fig. 5).

## Taurine and taurine derivatives in inflammatory diseases: their role in pathogenesis and treatment of MPO-associated chronic inflammation

The antimicrobial and anti-inflammatory properties of TauCl and TauBr make these agents good candidates for clinical use, especially for local treatment of infectious/inflammatory diseases (Gottardi and Nagl 2010; Marcinkiewicz 2009). So far, the therapeutic efficacy of these agents has been shown in acne vulgaris, external otitis, purulently coated crural ulcerations and keratoconjunctivitis (Gottardi et al. 2005, 2007; Marcinkiewicz et al. 2008; Nagl et al. 2000a, b, 2003; Neher et al. 2007). Moreover, it has been suggested that TauCl may be of potential benefit as adjunctive local therapy in periodontal diseases (Mainnemare et al. 2004). In contrast to the successful topical therapies mentioned above, rapid degradation of TauCl and TauBr in the blood limits their systemic application (Martini et al. 2012). As systemic therapy with taurine haloamines seems to be impossible, an alternative, novel strategy may be to administer taurine itself as a prodrug. Taurine supplementation may be predicted to enhance local formation of TauCl or TauBr, as exogenous taurine will react with endogenous HOCl/HOBr. Such strategies may be effective in inflammatory conditions associated with local infiltration of neutrophils, for example chronic sinusitis, inflammatory bowel disease and rheumatoid arthritis. So far, the beneficial effect of such strategy has been documented in experimental colitis treated with 5-aminosalicyltaurine (taurine conjugated with 5-ASA) (Kim et al. 2006; Joo et al. 2009), in dextran sulfate sodium (DSS)-induced experimental colitis in mice attenuated by dietary taurine supplementation (Shimizu et al. 2009) and in collagen-induced arthritis treated with taurolidine (Marcinkiewicz et al. 2006a, b). The precise mechanism underlying the beneficial effects of the dietary taurine supplementation is still unclear and remains to be explained.

In conclusion, both in vitro and clinical studies clearly indicate that both, taurine and taurine derivatives, may find their place in the therapy for various topical infections as well as chronic inflammatory diseases. In the next section, we will discuss the role of MPO–halide system products, namely TauCl in the pathogenesis of rheumatoid arthritis, the best studied model of inflammatory disease in our laboratories.

## Taurine and taurine derivatives in rheumatoid arthritis (RA) and collagen-induced arthritis (CIA)

Rheumatoid arthritis (RA) is a chronic systemic autoimmune disorder affecting approximately 1 % of the population and leading eventually to joint deformation, dysfunction and disability in most diseased individuals. The etiology and pathogenesis of RA are not fully understood. However, genetic (e.g., genes encoding HLA-DR molecules containing shared epitopes) and environmental factors (e.g., *Porphyromonas gingivalis* infection) are generally accepted as participating in disease development, while repeated activation of innate immunity and deregulated adaptive immunity are thought to contribute to inflammation chronicity and self-tolerance breakdown (Gregersen et al. 1987; Detert et al. 2010; Gierut et al. 2010; Scherer and Burmester 2011). Numerous cytokines, released primarily by cells that accumulate in the synovium (e.g., synoviocytes, infiltrating leucocytes), play a fundamental role in these pathological processes.

Novel biological therapies (cytokine antagonists, B cell depletion, T cell co-stimulatory blockers) markedly improved RA patients’ clinical outcomes, but impressive efficacy is only reached in about half of them (Scott 2012). Therefore, great efforts are made to indicate the new therapeutic targets.

### Pathological processes in rheumatoid arthritis joints

In a normal joint, the lining layer of the joint cavity (synovium) is composed of intimal lining and sublining formed by cells submerged in a bed of extracellular matrix (ECM). Macrophage-like (MfLS) and fibroblast-like (FLS) synoviocytes are present in the synovial intimal lining, where MfLS clear the joint from microorganisms and cellular debris, while FLS synthesize ECM and synovial fluid components. Rheumatic joints are characterized by synovial membrane inflammation (synovitis) and progressive damage to the articular cartilage and subchondral bone (Firestein 2009; Bartok and Firestein 2010). The number of MfLS and FLS rises dramatically and the intimal lining expands from 1–2 cells depth to a depth of up to 10–20 cells. Both types of rheumatoid synoviocytes display a highly activated phenotype and represent the major source of locally synthesized pro-inflammatory factors and enzymes degrading connective tissue. Moreover, synoviocytes form niches for infiltrating immune cells. By secretion of soluble factors as well as direct cell-to-cell interaction via adhesion molecules, synoviocytes support survival and differentiation of T and B lymphocytes into pathogenic Th17 subset and plasma cells, respectively. Lymphocytes and dendritic cells massively infiltrate the sublining layer and form ectopic lymphoid tissue, where autoantibodies are produced. Neutrophils pass through the synovium and accumulate in RA synovial fluid, where their number is extremely high, reaching up to 5 × 10^9^ cells. It is well known that neutrophils recruited into the site of inflammation generate a large number of highly reactive oxidants, including hypochlorous acid (HOCl). This highly reactive oxidant is immediately consumed and thus inactivated by reaction with the thiol groups of cellular proteins and proteins originating from engulfed pathogens, as well as by transferring of the active chlorine to amino groups. Neutrophils contain a large amount of taurine, which represents about 50 % of the cellular amino acid pool. Therefore, this dominant free amino acid is the key molecule able to trap HOCl. Reaction of HOCl with taurine results in the formation of taurine chloramine (TauCl), endowed with potent anti-inflammatory properties, as described above. Due to prolonged activation, neutrophils accumulating in rheumatoid synovial fluid exhibit features indicative of partial functional “exhaustion”. Importantly, these cells generate less TauCl in vitro than their peripheral blood counterparts, suggesting that the local concentration of TauCl in RA joints is probably too low to exert anti-inflammatory effects (Kontny et al. 2002). Thus, diminished local generation of TauCl may contribute to more complex immunoregulatory disturbances related to the chronic course of inflammatory response in RA joints.

As a result of the above events, rheumatoid synovium transforms to a hyperplastic, invasive tissue. At the cartilage–bone interface, this expansive tissue, called pannus, invades the cartilage and erodes into the bone. Due to their unique invasive properties and production of huge amount of proteases, FLS are the primary effectors of cartilage degradation. Progressive bone damage results from resorption of this tissue by osteoclasts and its inefficient restoration by osteoblasts (Schett et al. 2011).

We have recently reported that also rheumatoid articular adipose tissue (AAT) is also highly reactive and upon stimulation secretes considerable amounts of pro-inflammatory (IL-1β, IL-6, IL-8, TNF-α) and anti-inflammatory (IL-1 receptor antagonist—IL-1Ra) cytokines as well as classical adipokines (leptin, adiponectin). Moreover, we found this tissue to release biologically active factors that intensify the pathogenic activities of rheumatoid FLS. Thus, AAT should be considered a novel important contributor to the pathological processes taking place in the RA joints (Kontny et al. 2012).

### Taurine chloramine normalizes pathogenic functions of rheumatoid FLS

Among numerous factors secreted by FLS, VEGF and IL-8 recruit immune cells and support angiogenesis, PGE_2_ mediates vascular phase of inflammatory response and osteoclastic bone resorption, while IL-6 exerts pleiotropic effects, e.g., supports differentiation of T helper lymphocytes into Th17 subset, participates in bone loss and contributes to systemic symptoms. In vitro studies revealed that at physiologically relevant concentrations (200–500 μM), TauCl inhibits synthesis of these factors by several mechanisms: (1) acting at the transcriptional level, (2) diminishing DNA-binding activity of NFκB and AP-1 transcription factors or (3) up-regulating heme-oxygenase-1 (Kontny et al. 1999, 2000, 2003a, b, 2007; Muż et al. 2008). Another taurine derivative with potential immunoregulatory activity, taurine bromamine (TauBr), is less effective in normalization of these pro-inflammatory rheumatoid FLS properties (Kontny et al. 2007). Moreover, in these cells TauCl down-regulates also expression of collagenases (MMP-1, MMP-13) that play a dominant destructive role in RA (Kim et al. 2007, 2010a, b). Furthermore, TauCl inhibits proliferation of RA FLS and renders these cells more sensitive to death (Kontny et al. 2006a, b). Thus, in vitro TauCl dampens several activities of rheumatoid FLS relevant to the contribution of these cells to local pathological processes, i.e., inflammation support, joint destruction and synovial hyperplasia.

Interestingly, neither taurine alone nor sulfoacetaldehyde, a product of TauCl decomposition, exerts such suppressive effects on RA FLS (Kontny et al. 2003c). Thus, the unique activities of TauCl arise from its oxidative properties and selective modification of molecules implicated in cellular signal transduction pathways (Kontny et al. 2000; Kim et al. 2007; Muż et al. 2008).

### Taurine chloramine exerts immunomodulatory effect on the secretory activity of rheumatoid joint-associated adipose tissue

We have recently reported that not only synovium but also articular adipose tissue, organized into the largest Hoffa infrapatellar fat pad and three smaller fat pads, is a rich source of adipokines and other factors that participate in local pathological processes characteristic for RA (Kontny et al. 2012). In patients with osteoarthritis, articular adipose tissue is infiltrated by immune cells (monocytes, granulocytes, T lymphocytes) (Klein-Wieringa et al. 2011). The activity of cells present in **the** rheumatoid synovium is supported not only by locally produced cytokines and growth factors, but also by direct cell-to-cell interactions. Thus, the inhibitory effect of TauCl on isolated FLS does not entirely reproduce in situ circumstances. To mimic in vivo conditions, we examined the effect of TauCl on the secretory activity of these joint tissues. Tissue specimens of articular adipose tissue (AAT; *n* = 35–63), periarticular subcutaneous adipose tissue (ScAT; *n* = 19–25) and synovial membrane (SM; *n* = 17–25) were obtained from knee joint of patients (60F/3 M) with established RA (RA stages III–IV) at the time of total joint surgery, performed as a normal part of clinical care. Subcutaneous adipose tissue was taken from the site of the suture. All patients gave their informed consent and the study was approved by the Institute of Rheumatology Ethics Committee. The mean (range) patient’s age and disease duration was 55.3 (26–68) and 17 (6–34) years, respectively. Tissue preparation and cultures were performed as described previously (Kontny et al. 2012). Tissue explants (100 mg/ml) were cultured in Dulbecco’s modified Eagle medium (DMEM/50 μg/ml gentamicin/100 mg/ml kanamycin) alone, or stimulated for 18 h with 1 μg/ml of lipopolysaccharide from *Escherichia* *coli 055:B5* (LPS, Difco, Detroit, MI, USA) in the presence or absence of 500 μM TauCl. The concentrations of selected pro- and anti-inflammatory cytokines and classical adipokines in culture supernatants were measured by specific ELISA, as described previously (Kontny et al. 2012). IL-10 concentrations were measured using commercially available set (eBioscience, San Diego, CA, USA). Taurine chloramine (*N*-chlorotaurine sodium salt), was a gift from prof. Waldemar Gottardi and prof. Marcus Nagl from the Division of Hygiene and Medical Microbiology, Innsbruck Medical University, Austria. Data were analyzed using Statistica vol. 7.0 software. The Wilcoxon test was applied to evaluate the effect of LPS and LPS + TauCl. Differences were considered significant for *p < 0.05, **p < 0.01 and ***p < 0.001.

**Fig. 6:**
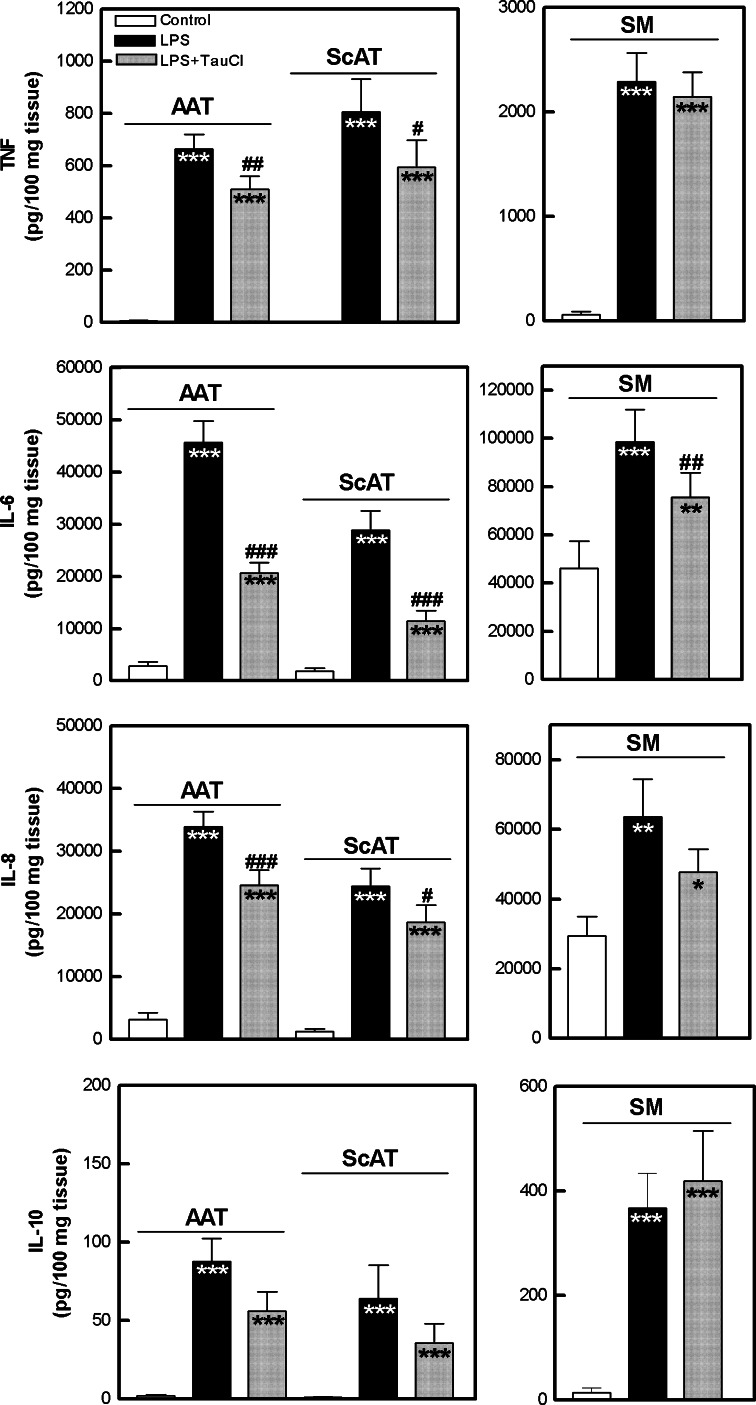
Effect of TauCl on pro- and anti-inflammatory cytokine release from articular adipose tissue (*AAT*), subcutaneous (*ScAT*) adipose tissue and synovial membrane (*SM*) explants. Tissue explants were cultured for 18 h in 37 °C in culture medium alone (control; *white bars*) or treated with LPS (1 μg/ml) in the absence (*black bars*) or presence of TauCl (500 μM) (*gray bars*), then cytokine concentrations in culture supernatants were measured by ELISA. Values are the mean and SEM of 35–53 (AAT), 20–25 (*ScAT*) or 14–24 (*SM*) experiments. *Indicates statistically significant differences between untreated and treated cultures; ^#^Indicates statistically significant differences between LPS− versus LPS+ TauCl-treated cultures; *^,#^ *p* < 0.05; **^,##^ *p* < 0.01; ***^,###^ *p* < 0.001

**Fig. 7:**
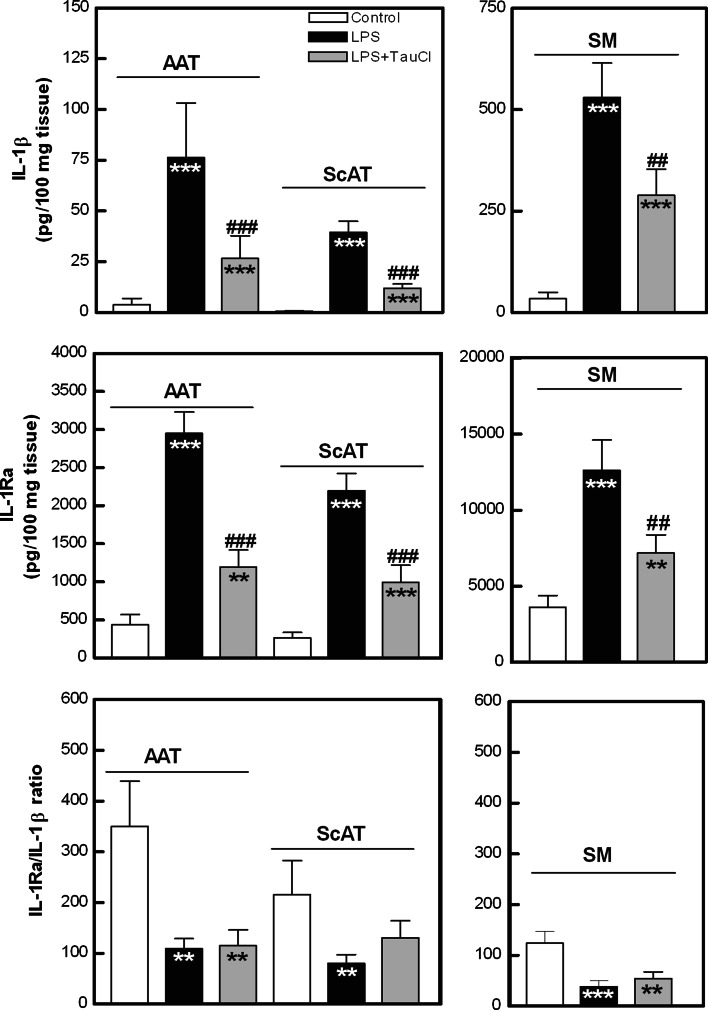
Effect of TauCl on IL-1β and IL-1Ra release from articular adipose tissue (*AAT*), subcutaneous (*ScAT*) adipose tissue and synovial membrane (*SM*) explants. Explanations as in Fig. 1. Values are the mean and SEM of 37–41 (*AAT*), 19–23 (*ScAT*) or 17–18 (*SM*) experiments

As shown in Figs. 6 and 7, both AAT and ScAT produced spontaneously smaller amounts of cytokines than SM, but cytokine release from all tested tissues rose significantly in the presence of LPS, known to activate cells via toll-like receptor 4. We used ScAT as a control tissue, but unexpectedly it responded to LPS treatment similarly to AAT, showing that adipose tissue, at least from these locations, was highly reactive to this pro-inflammatory stimulus. In LPS-treated adipose explant cultures, the addition of TauCl significantly inhibited the production of IL-6, TNF-α and IL-8 (% of inhibition was ≈60, 25 and 26 %, respectively). However, in equivalent SM cultures, only the secretion of IL-6 and IL-8 was diminished (by 23–25 %), but the reduction of IL-8 release did not reach statistical significance. By contrast, in all tissue cultures, LPS-triggered release of anti-inflammatory IL-10 was not significantly affected upon TauCl treatment (Fig. 6).

Interleukin 1β is another pro-inflammatory cytokine which plays a critical role in RA pathogenesis. The biological activity of IL-1β is counteracted by IL-1Ra, but for successful blockade a large molecular excess (100-fold to 1,000-fold) of IL-1Ra is required (Gabay et al. 2010). As shown in Fig. 7, all tested tissues secreted spontaneously higher amounts of IL-1Ra than IL-1β. However the IL-1Ra/IL-β ratio was higher in AAT and ScAT than in SM cultures, suggesting that in adipose tissue IL-1β activity is better controlled. In all tissue cultures, LPS markedly elevated both IL-1β and IL-1Ra release, leading to a dramatic decrease of IL-1Ra/IL-1β ratio. In the presence of TauCl, significantly reduced secretion of both IL-1β and IL-1Ra was observed and thus IL-1Ra/IL-1β ratio was not restored (Fig. 7).

It has been suggested that also classical adipokines also participate in RA pathogenesis, but their role is still controversial. Leptin is structurally classified as a member of the long-chain helical cytokine family, which includes several pro-inflammatory cytokines, e.g., IL-6 and IL-12. Both pro- and anti-inflammatory properties of leptin have been described (Stofkova 2009; Neumann et al. 2011). Adiponectin structurally belongs to the collagen superfamily and shares homologies with the collagens, complement factors and TNF-α. Adiponectin has effects in a number of different tissues, e.g., it counteracts insulin resistance in muscle, reduces atherosclerosis, as well as prevents the deleterious effects of TNF-α on endothelial cells by reducing adhesion molecule expression and inflammation (Gustafson 2010). In contrast in RA, adiponectin appears to demonstrate mostly pro-inflammatory and pro-destructive effects (Frommer et al. 2010; Neumann et al. 2011). Adiponectin exists in several forms (globular and multimers of high and low molecular weight) that differ in biological activities (Gustafson 2010). Unfortunately, the relevance of these adiponectin forms to RA pathological processes is unknown.

**Fig. 8:**
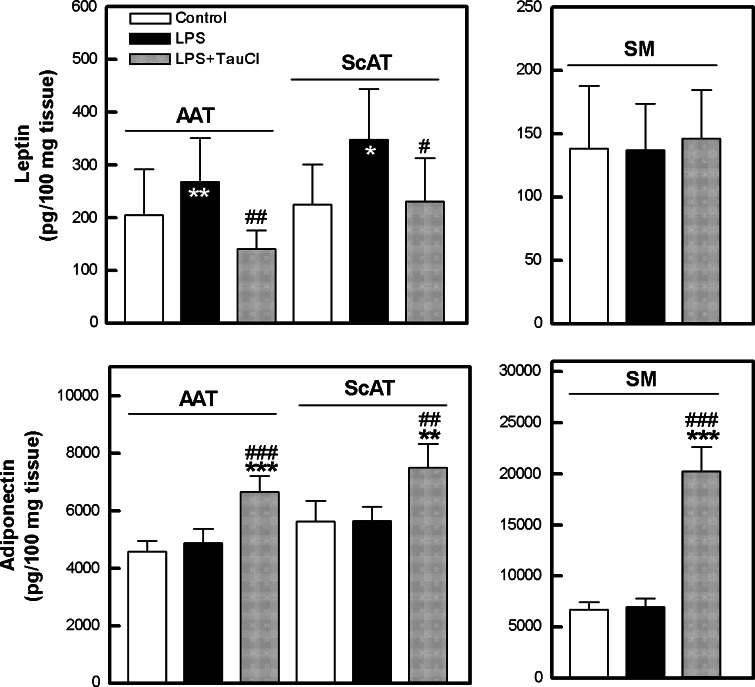
Effect of TauCl on the release of classical adipokines from articular adipose tissue (*AAT*), subcutaneous (*ScAT*) adipose tissue and synovial membrane (*SM*) explants. Explanations as in Fig. 1. Values are the mean and SEM of 54 (*AAT*), 20–23 (*ScAT*) or 25–28 (*SM*) experiments

Interestingly, the expression and release of adipokines is reciprocally regulated by inflammatory stimuli. Acute inflammation and pro-inflammatory cytokines (TNF-α, IL-1, IL-6) positively regulate leptin expression and its circulating levels, whereas long-term exposition to IL-1 or TNF-α exerts negative effects. By contrast, the same pro-inflammatory cytokines are potent inhibitors of adiponectin gene expression or protein secretion (Stofkova 2009). As shown in Fig. 8, spontaneous secretion of leptin and adiponectin from all tested tissues was similar. In adipose explant cultures, LPS significantly up-regulated leptin release, and addition of TauCl counteracted the LPS effect, while in SM cultures this adipokine secretion was not affected by the treatment. By contrast to leptin, LPS failed to exert any effect on the release of adiponectin. However, in the presence of TauCl, all tissues secreted significantly more adiponectin than both untreated or LPS-treated explants.

Based on the above findings, we report for the first time that TauCl is a potent inhibitor of LPS-triggered pro-inflammatory cytokine (IL-1β, IL-6, IL-8, TNF-α) secretion by joint-associated adipose tissues. Although TauCl exerted weaker inhibitory effect on SM secretory activity, suggesting that this compound has limited capability for termination of synovitis, down-regulation of IL-6 and IL-1β was observed. This is an important finding, as TNF-α, IL-1β and IL-6 all play a critical role in RA pathogenesis and are potent activators of cells present in SM. For this reason, therapy of RA by neutralization of these cytokines by biological drugs is being pursued (Scott 2012). Unfortunately, TauCl reduced IL-1Ra release. Due to this effect, residual IL-1β could not be controlled efficiently. However, in all tested tissues, TauCl did not inhibit the production of IL-10, a cytokine of known anti-inflammatory properties. Therefore, it is likely that the net local effect of TauCl is anti-inflammatory. Moreover, TauCl modifies classical adipokine secretion by inhibiting leptin and enhancing adiponectin release. As the role of these adipokines in RA pathology is far from clear, the net effect of this TauCl action on local pathological processes is unpredictable at the moment. Nevertheless, the present results expand the spectrum of known anti-inflammatory activities of TauCl and give further support to considering this compound as a promising candidate for RA treatment. However, it should be underlined that the above effects observed upon TauCl treatment cannot be attributed only to the direct action of this compound, because TauCl may react with the culture medium components, resulting in the formation of other chloramines. Thus, the contribution of the newly formed chloramines and their decomposition products cannot be excluded.

### Taurine chloramine in collagen-induced arthritis (CIA)

There are reports showing that administration of TauCl improves the course of arthritis in various experimental animal models. Therapeutic benefits have been ascribed to both anti-inflammatory and connective tissue protective action of this compound. For example, intraperitoneal TauCl administration modified adjuvant-induced arthritis in rats due to down-regulation of the inflammatory mediators (histamine and oxygen radical species) generation (Wojtecka-Łukasik et al. 2005). On the other hand, in septic arthritis induced by intra-articular injection of a single dose of *Staphylococcus aureus*, locally administered TauCl exerted an inhibitory effect on the development of bone and cartilage damage in the infected joint, but no beneficial effects were observed when bacteria and TauCl were administered systemically (Verdrengh and Tarkowski 2005). Collagen-induced arthritis (CIA) is an experimental model of RA, studied extensively to elucidate the pathogenic mechanisms of the disease and to identify potential therapeutic targets. In genetically susceptible (DBA 1/J) mice, the disease can be induced by immunization with native type II collagen in adjuvant. We have previously shown that systemic administration of TauCl either delayed CIA onset or significantly reduced the incidence of the disease, depending on whether TauCl therapy is applied early (after primary immunization) or late (after booster immunization) during the CIA course, respectively (Kwaśny-Krochin et al. 2002). Thus, we concluded that systemic application of TauCl could not alleviate the symptoms of arthritis, but may prevent CIA development. Recently, others (Wang et al. 2011) have reported that TauCl administered in the same way significantly attenuated the severity of CIA symptoms, i.e., synovial inflammation, cartilage damage and bone erosion. Moreover, in the joints of TauCl-treated mice, the number of osteoclasts was reduced and in vitro TauCl inhibited osteoclastogenesis, supporting the idea that this compound exerts protective effect on the bone. Although the precise mechanisms underlying TauCl inhibition of CIA are not fully understood and require further studies, the above data support the proposal that TauCl may be a useful candidate for complementary arthritis treatment. However, to improve therapeutic effectiveness, the stability of TauCl should be increased. Recently, C-methylated derivatives of TauCl with better stability at room temperature have been obtained (Low et al. 2009; Shiau et al. 2008). In addition, a synthetic derivative of taurine—bis(1,1-dioxoperhydro-1,2,4-thiabiazin-4-yl)methane, named taurolidine (TRD)—is another potential candidate for RA therapy. Owing to its bactericidal, anti-inflammatory, antiangiogenic and antitumor properties, TRD has been used in the treatment of patients with peritonitis, sepsis or gastrointestinal and nervous system tumors (Schneider et al. 2005; Willatts et al. 1995; McCourt et al. 2000). In several European countries, TRD is currently licensed for intraperitoneal use for the treatment of peritonitis, and clinical trials evaluating TRD potential antineoplastic benefits are currently underway (Neary et al. 2010). The mechanism of TRD action is not fully understood. Taurolidine is in vivo degraded to methylol-containing products, which exert antibacterial, antiendotoxin and antiadherence activities, and taurine, which is devoid of such properties. It is likely that in vivo TRD-originated taurine may react with HOCl to produce anti-inflammatory TauCl, but TRD can exert anti-inflammatory effect also by other mechanisms (Willatts et al. 1995; Marcinkiewicz et al. 2006a, b; Neary et al. 2010). Importantly, intraperitoneal treatment with TRD reduced the incidence of CIA in mice, while intra-articular application of TRD resulted in amelioration of ovalbumin-induced arthritis in rabbits (Marcinkiewicz et al. 2007).

## Summary

- The fundamental role of taurine in the immune system is related to its antioxidant properties. Taurine protects tissues from oxidative stress associated with the pathology of various inflammatory diseases.
- Taurine, the component (or modulator) of the myeloperoxidase–halide system of leukocytes, reacts with HOCl/HOBr to produce taurine haloamines (TauCl/TauBr), which are less toxic milder oxidants, but retain antimicrobial and anti-inflammatory properties.
- Taurine and taurine haloamines are components of innate immunity. The physiological functions of TauCl/TauBr are associated with the MPO–halide system of neutrophils.

In conclusion, both in vitro and in vivo studies as well as clinical trials give support to consider taurine and taurine derivatives as potential drugs in human medicine, including infectious and chronic inflammatory disease. However, further studies are necessary to improve their therapeutic effectiveness, especially in the treatment of biofilm-associated infections.
